# Letter to the Editor

**DOI:** 10.1120/jacmp.v12i1.3530

**Published:** 2010-12-14

**Authors:** Michael S. Gossman, A. Jussi Pahikkala, Mary B. Rising, Patton H. McGinley

Dear Editor‐in‐Chief,

We write this letter in reference to our article; Gossman et al. “*Providing solid angle formalism for skyshine calculations*,” *J. Appl. Clin. Med. Phys. 2010; 11(4): 278–282.* ^(^ ^1^ ^)^ As a result of numerous private requests for us to provide an additional equation for the solid angle used in skyshine calculations for machines which have non‐circular and non‐square open apertures, we have elected to satisfy this need.^(^ ^2^ ^)^ The additional equation for solid angle is applicable for skyshine calculations involving some Elekta machines (Precise Treatment System™, Elekta Synergy®, and Elekta Synergy® Platform), which we understand to have a Beam Modulator that opens maximally to a field‐size specification of 16 cm× 21 cm.

For discussion here, we define the distance *h* as the height of the beam for which the solid angle is determined. Given this geometry, a rectangular radiation beam aperture will project an inverted rectangular pyramid with SAD‐defined field size denoted a×b. Conclusively, the form of the resulting equation for the solid angle that is used for medical accelerator skyshine calculations is now presented as Eq. (1):

(1) ΩRectangle=4arcsin=ab(a2+4h2)(b2+4h2)

It is important to use the correct equation based on the shape of the collimating aperture from which the radiation is emitted. We now remind the reader of the form of the solid angle equations for other aperture shapes in Eq. (2) and in Eq. (3):

(2) $$\Omega_{\mathrm{Square}}=4\text{arcsin}=\frac{a^{2}}{a^{2}+4h^{2}}$$

(3) $$\Omega_{\mathrm{circle}}=2\pi (1-\text{cos}\theta )$$

Note that Eq. (1) provides the same results as Eq. (2) when the field sizes in both directions are the same (a=b). Also, in Eq. (3), angle θ (degrees) is defined as the angle subtended between the central axis and the edge of the beam as radiation projects away from the source for circular apertures. These equations may be used for machines other than the Elekta model stated here or within the referenced research. Also, the equations are not limited to skyshine for bremsstrahlung photon irradiations. These should also be used when dealing with gamma producing brachytherapy (i.e., ${\;}^{192}\text{Ir}$) and teletherapy (i.e., ${\;}^{60}\text{Co}$) radiation sources, as well as for neutron skyshine calculations.^(^ ^3^ ^,^ ^4^ ^)^

Sincerely,
